# Human Activity Recognition via Score Level Fusion of Wi-Fi CSI Signals

**DOI:** 10.3390/s23167292

**Published:** 2023-08-21

**Authors:** Gunsik Lim, Beomseok Oh, Donghyun Kim, Kar-Ann Toh

**Affiliations:** 1 School of Electrical and Electronic Engineering, Yonsei University, Seoul 03722, Republic of Korea; gslim@yonsei.ac.kr (G.L.); kimd@yonsei.ac.kr (D.K.); 2 Department of Applied Artificial Intelligence, Seoul National University of Science and Technology, Seoul 01811, Republic of Korea; bsoh@seoultech.ac.kr

**Keywords:** human activity recognition, Wi-Fi CSI signals, score-level fusion

## Abstract

Wi-Fi signals are ubiquitous and provide a convenient, covert, and non-invasive means of recognizing human activity, which is particularly useful for healthcare monitoring. In this study, we investigate a score-level fusion structure for human activity recognition using the Wi-Fi channel state information (CSI) signals. The raw CSI signals undergo an important preprocessing stage before being classified using conventional classifiers at the first level. The output scores of two conventional classifiers are then fused via an analytic network that does not require iterative search for learning. Our experimental results show that the fusion provides good generalization and a shorter learning processing time compared with state-of-the-art networks.

## 1. Introduction

Human activity recognition (HAR) is a field of research and technology that focuses on developing methods for automatically identifying and understanding human activities using sensor data [1,2]. HAR has a wide range of applications in various domains, including healthcare, security, sports, robotics, and surveillance (see e.g., [1,3]). In recent years, the importance of HAR has grown significantly due to its potential benefits and the many practical applications it offers [4].

There are three main approaches to HAR: vision-and-sound-based [5], mobile-device-based [6], and Wi-Fi-signal-based [7]. Each approach has its own advantages and challenges. The vision-and-sound-based approach can raise concerns about security and privacy due to the use of cameras and microphones. The mobile-device-based approach requires individuals to wear or carry smart devices, which can be expensive and inconvenient. In contrast, the Wi-Fi-based approach does not involve the use of cameras or microphones, so it does not raise the same concerns about security and privacy [8]. Additionally, individuals do not need to wear or carry any devices, as Wi-Fi signals are readily available in most environments. This makes the Wi-Fi-based approach a promising candidate for HAR. A brief review is provided in Section 2 regarding each of these approaches.

Wi-Fi signals, which operate within the radio frequency spectrum, can be affected by various factors such as interference, obstructions, and signal absorption. When humans move or interact with objects within the signal path, they can inadvertently affect the propagation of Wi-Fi signals [9]. This can lead to observable patterns in signal quality and connectivity. For example, the human body itself can act as an obstacle that attenuates or blocks Wi-Fi signals. When a person moves around a space, their physical presence can cause fluctuations in signal strength as the signals encounter the obstruction posed by their body. These disturbances can manifest as recognizable patterns in Wi-Fi connectivity, often seen as intermittent drops or variations in signal strength [7]. By capitalizing on this phenomenon, it is possible to use disturbed Wi-Fi CSI signals for HAR.

Both classical machine learning and deep learning methods have been used for HAR. Classical machine learning methods, such as Principal Component Analysis (PCA) (see e.g., [10]), K-Nearest Neighbors (KNN) (see e.g., [11]), and Support Vector Machines (SVM) [12], have the advantage of having low training demands but may compromise recognition accuracy. On the other hand, deep learning methods, such as Convolutional Neural Networks (CNN), Long Short-Term Memory (LSTM), and Gated Recurrent Unit (GRU) [13], offer high recognition accuracy but require large amounts of data and significant training effort. Unlike face recognition, where there is a large collection of images available from social media, HAR using Wi-Fi signals does not have a large enough dataset to train a deep network effectively. This has motivated the study of fusion structures that balance sample size, training effort, and prediction accuracy.

The main contribution of this work is the development of a fast-learning fusion network that avoids over-fitting while using a relatively small number of training samples. This approach balances learning accuracy with training sample size. To evaluate the effectiveness of the preprocessing and fusion settings, an extensive series of experiments were conducted on three publicly available datasets.

The paper is structured as follows: Section 2 offers a concise overview of related works. Section 3 and Section 4 introduce the preliminaries and our proposed study for HAR. In Section 5, we present our experimental setup and results with discussion. Finally, Section 6 presents our conclusion for the study.

## 2. Related Work

### 2.1. Vision-and-Sound-Based HAR

For vision-and-sound-based systems, a comprehensive survey of space–time and hierarchical approaches for HAR is provided by Aggarwal et al. [1]. In video-based systems, moving pictures are converted into 2D images, and image features are extracted for classification. For example, Anitha et al. [14] developed a system to recognize hand gestures by converting human action videos into 2D images and extracting features using Laplace Smoothing Transform (LST) and Kernel Principal Component Analysis (KPCA), with KNN used for classification. Local space–time features can also be used with classifiers such as SVM for recognizing human actions [15]. Ahmad et al. [16] used Spatio-Temporal Interest Points (STIPs) to detect important changes in images, extracting appearance and motion features using Histogram of Oriented Gradient (HOG) and Histogram of Optical Flow (HOF) descriptors, with SVM used for classification. Since using videos and images can jeopardize user privacy, other non-revealing sensors have been explored for HAR. For instance, Fu et al. [17] developed a motion detector with sub-millisecond temporal resolution using a contrast vision sensor. Sound signals have also been used, such as in the work of Stork et al. [18], who proposed a Non-Markovian Ensemble Voting (NEV) method to classify multiple human activities in real-time based on characteristic sounds. Additionally, 3D skeletal data have been utilized, as in the work of Ramezanpanah et al. [19], who represented 3D skeletal features of human action using Laban movement analysis and dynamic time warping, with SVM used for activity classification.

In addition to the classical machine learning methods mentioned above, recent work has focused on using deep learning for human action or activity recognition. For example, Wang et al. [20] developed a system for 3D human activity recognition using a re-configurable Convolutional Neural Network (CNN). Other examples of deep learning-based applications include the work of Dobhal et al. [21], who recognized human activity based on binary motion images and deep learning. In the work of Mahjoub et al. [22], image sequences were combined into a single Binary Motion Image (BMI) for feature representation, with a CNN used for classification.

### 2.2. Mobile-Device-Based HAR

In healthcare systems, wearable sensors are commonly used to recognize human activities. According to a survey by Thakur et al. [2], smartphone sensors such as accelerometers, gyroscopes, magnetometers, digital compasses, microphones, GPS, and cameras can be used to monitor and recognize human activities. For example, Anjum et al. [23] developed a smartphone application that uses the embedded motion sensor to track users’ physical activities and to provide feedback. The application estimates calories burned and breaks it down by activity such as walking, running, climbing stairs, descending stairs, driving, cycling, and being inactive. Another example is a framework proposed by Nandy et al. [24] that combines features from a smartphone accelerometer and a wearable heart rate sensor to recognize intense physical activity. The framework uses an ensemble model based on different classifiers. In addition to smartphone sensors, wearable acoustic sensors such as the bodyscope developed by Yatani et al. [25] can be used to detect and classify human activities. Human activities can also be remotely monitored via the use of wearable sensors that track heart rate, respiration rate, and body acceleration. Castro et al. [26] developed a remote monitoring system based on the Internet of Things (IoT) that uses these sensors.

The development of mobile-device-based HAR has followed a similar trend to vision-and-sound-based HAR in terms of the popularity of deep learning deployment. In addition to MLP and CNN, LSTM is also a popular choice. For example, Voicu et al. [27] used smartphone sensors such as accelerometers, gyroscopes, and gravity sensors to recognize physical activity, with an MLP used for learning and classification. Rustam et al. [28] employed sensor data from gyroscopes and accelerometers with a deep network model called Deep Stacked Multilayered-Perceptron (DS-MLP) for HAR. Chen et al. [29] constructed a CNN network for HAR based on a single accelerometer, with modified convolution kernels to adapt to the characteristics of tri-axial acceleration signals. Ghate et al. [30] constructed a hybrid deep learning model that combines deep neural networks with LSTM and GRU for effective classification of engineered features of CNN. The network integrates CNN with a Random Forest Classifier (DeepCNN-RF) to add randomness to the model.

### 2.3. Wi-Fi-Based HAR

Different from the mobile-based approach, the Wi-Fi signals offer a device free solution for HAR. Compared with vision-based methods, the Wi-Fi signals do not provide detailed information related to privacy. There are mainly two types of Wi-Fi signals commonly used for HAR: the RSSI and the CSI signals. The RSSI refers to a coarse-grained received signal strength indicator, and the CSI refers to the fine-grained channel state information. The RSSI is susceptible to signal fading, distortions, and inconsistency since it has a low resolution parameter, which is measured using a packet index and has only a single value per packet. For this reason, the RSSI is being replaced by the CSI in Wi-Fi sensing solutions. The Wi-Fi CSI is a fine grained signal measured via Orthogonal Frequency Division Multiplexing (OFDM) subcarriers. The following review has been conducted according to these two types of Wi-Fi technology.

In terms of using RSSI signals, four wireless technologies—Wi-Fi, BLE, Zigbee, and LoRaWAN—have been evaluated for indoor localization. According to a study by Sadowski et al. [31], Wi-Fi was found to be the most accurate among the investigated wireless technologies based on RSSI signals. In another example, Hsieh et al. [32] recognized human activity in an indoor environment using Wi-Fi RSSI and investigated the effectiveness of several machine learning methods, such as MLP and SVM, for activity detection.

In terms of using CSI signals, it has been found that CSI can capture unique patterns of small-scale fading caused by different human activities at a subcarrier level, which is not available in traditional received signal strength (RSS) extracted at the per-packet level [33]. Most methods that use CSI signals employ deep networks. For example, Chen et al. [34] constructed a deep learning network for HAR that uses an attention-based bi-directional long short-term memory model. Wang et al. [35] developed a deep learning network that combines hidden features from both temporal and spatial dimensions for accurate and reliable recognition. Other examples include the work of Lu et al. [36] and Islam et al. [37], who implemented a channel-exchanging fusion network to fuse CSI amplitude and phase features for HAR and constructed a spatio-temporal convolution with nested long short-term memory (STC-NLSTMNet) to extract spatial and temporal features concurrently for automatic recognition of human activities, respectively. Recent algorithms such as transfer learning and attention mechanisms have also been used in HAR. For instance, Yang et al. [38] used an attention mechanism with LSTM features at different dimensions for HAR, while Jung et al. [8] performed in-air handwritten signature recognition using transfer learning due to limited data availability.

Finally, we shall focus on the use of CNN, a popular network for CSI-based HAR. In the work of Moshiri et al. [39], CSI data were converted into images and a 2D CNN classifier was employed for HAR. Zhang et al. [40] exploited semantic activity features and temporal features from different dimensions to characterize activity at different locations. The semantic activity features were extracted using a CNN combined with a convolutional attention module (CBAM), while the temporal features were extracted using a Bidirectional Gated Recurrent Unit (BGRU) combined with a self-attention mechanism. In addition to semantic features, dimension reduction techniques have also been used with CNN for HAR. Showmik et al. [41] proposed a Principal Component-based Wavelet Convolutional Neural Network (PCWCNN), which uses PCA and Discrete Wavelet Transform (DWT) as preprocessing algorithms and a Wavelet CNN for classification. Zou et al. [42] fused a tailored CNN model with a variant of the C3D model using vision for HAR.

Summarizing the above work, it is noted that vision-and-sound-based approaches pose the fundamental limitations in terms of securing the audio and video passages in view of the potential violation of human privacy.

## 3. Preliminaries

### 3.1. Wi-Fi for Human Activity Recognition

As mentioned in the section of related work, the Wi-Fi RSSI and the Wi-Fi CSI are two types of signal measurements that can be utilized for HAR [7]. The principle underlying these technologies is the Doppler effect, where the wavelength of reflected signals changes according to different relative motions of the human under the signal propagation space [43]. The RSSI stands for received signal strength indicator, which is an energy characteristic of the Media Access Control layer [44]. However, due to its reliance on channel strength superposition, it may not accurately reflect changes in the channel, leading to a reduced detection rate. On the other hand, the CSI represents a fine-grained channel state information, which includes specific indicators such as carrier signal strength, amplitude, phase, and signal delay [45]. The physical layer of CSI can capture micro dynamic changes in human activity, leading to the ability to detect rapid changes caused by the superposition of multipath signal exchange layers. Conceptually, the channel response is the response to RSSI, similar to how the rainbow responds to solar beams. We can think of OFDM as the medium that refracts RSSI into CSI, allowing the components of different wavelengths to be separated. As a result, the OFDM modulation system can make Wi-Fi-based HAR systems more robust against complex indoor environments, improving their effectiveness and accuracy [46].

### 3.2. Wi-Fi CSI Signal Features

The CSI is the channel response extracted from the OFDM subcarriers using fine-grained wireless channel measurement [7,45,47]. In an OFDM system, the CSI data on each subcarrier are modulated and converted into frequency domain via Fast Fourier Transform [46]. This provides an estimate of the amplitudes and phases of each subcarrier of the channel properties. During operation, the signal that is transmitted experiences multiple paths before arriving at the receiver. Each of these paths introduces distinct variations in time delay, amplitude attenuation, and phase shift where the Channel Impulse Response (CIR) [46] can be expressed as

(1) $$h\left(t\right)=\sum_{i=1}^{n}a_{i}e^{-j\theta_{i}}\delta \left(t-\tau_{i}\right).$$

In this equation, a signal from the *i*th path is represented by $a_{i}$ for its amplitude, $\theta_{i}$ for its phase, and $\tau_{i}$ for its time delay. *n* denotes the total number of paths, and δ(t) refers to the Dirac delta function.

For data collection in practice, the Channel Frequency Response (CFR) can be utilized to model the transmitting channel in place of the CIR. This is because the commodity hardware may not have the required time resolution to capture rapid changes in the signal. Under the unlimited bandwidth condition, the CFR can be derived from the CIR by applying the Fast Fourier Transform.

In frequency domain, the channel response of each subcarrier can be written as

(2) $$H\left(f_{k}\right)=\left|H\left(f_{k}\right)\right|e^{j∠H(f_{k})},$$

for k=1,…,K, where $H(f_{k})$ denotes the CSI of the *k*-th subcarrier, and $∠H(f_{k})$ represents the corresponding phase shift information. By packing the CSI data based on the subcarrier index and the packet number, we can write

(3) $$H=\left[\begin{array}{cccc} H^{1,1} & H^{1,2} & \ldots & H^{1,P}\\ H^{2,1} & H^{2,2} & \ldots & H^{2,P}\\ \vdots & \vdots & \ddots & \vdots \\ H^{K,1} & H^{K,2} & \ldots & H^{K,P} \end{array}\right],$$

where {1,2,…,K} denotes the subcarrier indices and {1,2,…,p} denotes the packet numbers.

## 4. Proposed System

**Figure 1:**
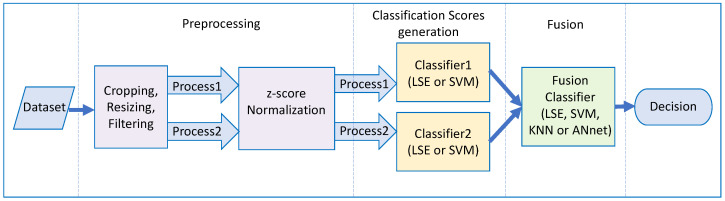
Pipeline of the fusion system.

In this study, we adopt the score fusion strategy to learn and predict the class labels of human activities. Figure 1 shows the pipeline of the implemented system. Essentially, the raw data of Wi-Fi CSI signals go through a preprocessing stage for normalization. The differently normalized data, process1 and process2, are then classified separately via a linear model based on the linear Least Squares Error (LSE) and a nonlinear model based on the SVM utilizing the Radial Basis Function (RBF) kernel. The learned scores are subsequently concatenated to form the input features for fusion learning. The fusion learning uses ANnet, or another LSE classifier, KNN classifier, or SVM-RBF classifier for a final decision. The final decision has been based on the one-versus-rest technique.

### 4.1. Preprocessing

In view of the noisy nature, the raw Wi-Fi CSI signals are preprocessed before being fed into the learning algorithms. Firstly, the signals are cropped according to each activity at different lengths. Since the action activities have different lengths, the CSI signals are resized based on linear interpolation. The resized data are then packed in matrix form for further processing prior to learning and prediction. Subsequently, a low-pass filtering has been performed to remove high frequency noises. Eventually, a *z*-score normalization is formed to remove the signal bias.

**Figure 2:**
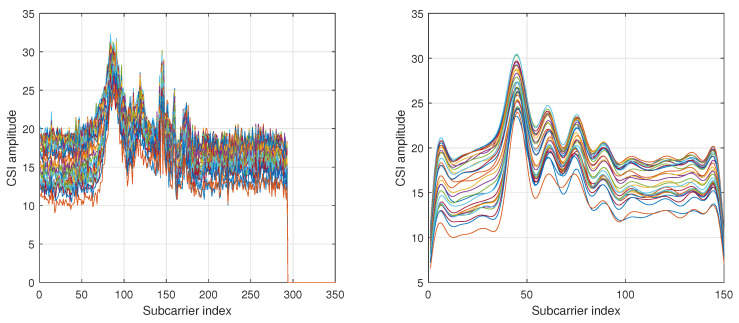
(**Left**) CSI samples before preprocessing. (**Right**) CSI samples after cropping, resizing, and filtering. Each colored line represents a sample sequence.

**Figure 3:**
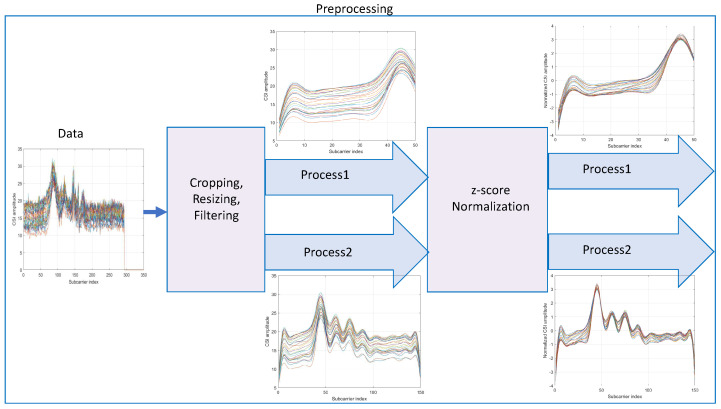
Pipeline of preprocessing steps.

The pre-processed signals are packed as shown in (4) for a subsequent stage of learning and prediction.

(4) $$H_{processed}=\left[\begin{array}{cccc} H^{1,1} & H^{1,2} & \ldots & H^{1,C}\\ H^{2,1} & H^{2,2} & \ldots & H^{2,C}\\ \vdots & \vdots & \ddots & \vdots \\ H^{K,1} & H^{K,2} & \ldots & H^{K,C} \end{array}\right],$$

where k∈{1,2,…,K} denotes the subcarrier index and *C* denotes the cropping size of the preprocessing. Figure 2 shows a sample of CSI raw signals and the preprocessed form before *z*-score normalization. The flow of the preprocessing steps is summarized in Figure 3. As illustrated in this figure, a set of cropped signals at length1 is named as process1 while another set of signals cropping at length2 is named as process2. The signals of process1 and process2 are eventually standardized via z-score normalization in the preprocessing step.

### 4.2. Classification Stage

The matrix for each activity sample $H_{processed}$ is flattened to form a row vector $h^{T}\in R^{1\times D}$ so that the *M* training samples can be stacked in matrix form as follows:

(5) $$X=\left[\begin{array}{c} h_{1}^{T}\\ \vdots \\ h_{N}^{T} \end{array}\right]\in R^{M\times D}.$$

Correspondingly, the *K* number of target activities is encoded based on the one-hot encoder such as

(6) $$Y=\left[\begin{array}{cccc} 1 & 0 & \cdots & 0\\ 1 & 0 & \cdots & 0\\ 0 & 1 & \cdots & 0\\ 0 & 1 & \cdots & 0\\ \vdots & \vdots & \ddots & \vdots \\ 0 & 0 & \cdots & 1 \end{array}\right]\in R^{M\times G},$$

where each sample row contains a ‘1’ at the column position corresponding to the class label. For first-level activity classification, two base classifiers namely the LSE and the SVM utilizing the RBF kernel have been deployed.

For training the linear prediction model Y=XW based on LSE, the learning weights $W\in R^{D\times G}$ can be found deterministically as

(7) $$\hat{W}=\left\{\begin{array}{cc} {\left(X^{T}X+\lambda I\right)}^{-1}X^{T}Y, & \mathrm{if}\;M>=D\\ X^{T}{\left(XX^{T}+\lambda I\right)}^{-1}Y, & \mathrm{if}\;M<D \end{array}\right.$$

with regularization λ=0.001 and consideration of over-/under-determined systems. Subsequently, the prediction of test samples can be computed using

(8) $$\hat{Y}=X_{t}\hat{W},$$

where $X_{t}\in R^{N\times D}$ denotes the test matrix. These output scores will be used in the subsequent stage of fusion for final prediction.

For binary classification, the SVM learning can be written as

(9) $$\mathrm{minimize}:\frac{1}{2}{\left||w|\right|}^{2}+C\sum_{i=1}^{n}\xi_{i},$$

(10) $$\mathrm{subject}\;\mathrm{to}:y_{i}\left(\phi {\left(x_{i}\right)}^{T}w+b\right)\geq 1-\xi_{i},$$

where ϕ corresponds to the RBF function, and $x_{i}$ and $y_{i}$ are the *i*th sample of input feature vector and target value, respectively. For multiclass problems, multiple SVMs can be implemented with the one-versus-rest technique for class prediction. In our study, the output probability score values of multicategory SVM are used as input features in the fusion stage.

### 4.3. Fusion Stage

We represent, respectively, the prediction scores obtained from the first level LSE and SVM as ${\hat{Y}}_{\mathrm{LSE}}\in R^{M\times G}$ and ${\hat{Y}}_{\mathrm{SVM}}\in R^{M\times G}$, where *M* denotes the sample size and *G* denotes the number of activity categories. Then, the scores for fusion can be stacked as

(11) $$F_{\mathrm{scores}}=\left[{\hat{Y}}_{\mathrm{LSE}},{\hat{Y}}_{\mathrm{SVM}}\right]\in R^{M\times 2G}.$$

According to [48], a network of two layers with sufficient hidden nodes can learn well data samples of limited size. Here, we implement a two-layer network known as ANnet [48] given by

(12) $$Y=\phi \left(\phi \left(F_{\mathrm{scores}}\right)W_{1}\right)W_{2}$$

to learn the stacked output scores (11) from the first-level LSE and SVM classification. In our implementation, the arctan ($\tan^{-1}$) function has been adopted as the nonlinear transformation ϕ. Similar to that in LSE, the learning target Y can adopt the one-hot encoding where the weights can be learned based on

(13) $$\begin{array}{ccc} {\hat{W}}_{1} & = & \phi {\left(F_{\mathrm{scores}}+\gamma N\left(k\right)\right)}^{†}, \end{array}$$

(14) $$\begin{array}{ccc} {\hat{W}}_{2} & = & \phi {\left(\phi \left(F_{\mathrm{scores}}+\gamma N\left(k\right)\right){\hat{W}}_{1}\right)}^{†}Y, \end{array}$$

where † denotes the Moore–Penrose inverse, which has been implemented using

(15) $$A^{†}=\left\{\begin{array}{cc} {\left(A^{T}A+0.01I\right)}^{-1}A^{T}, & \mathrm{if}\;M>=D\\ A^{T}{\left(AA^{T}+0.01I\right)}^{-1}, & \mathrm{if}\;M<D \end{array}\right.\;\;\;\;,\;A\in R^{M\times D}$$

considering stability of inversion in Python. In this learning, a random perturbation $\gamma R\left(k\right)\in R^{M\times 2G}$, where R is a random matrix, has been included to spread the data. The scaling factor γ∈R of the perturbation and the random perturbation seed k∈N can be considered as hyperparameters to be determined based on cross-validation utilizing the training set.

For prediction using unseen $F_{\mathrm{testscores}}$, the learned network weights can be substituted into (12) to obtain the estimated scores:

(16) $$\hat{Y}=\phi \left(\phi \left(F_{\mathrm{testscores}}\right){\hat{W}}_{1}\right){\hat{W}}_{2}.$$

The one-versus-rest technique can be applied to obtain the class label prediction.

## 5. Experiments

### 5.1. Database

Three datasets have been utilized for our experimentation. The first CSI dataset, which we called HAR-RP, has been obtained from [39]. This dataset contains seven different activities including RUN, WALK, FALL, BEND, SIT DOWN, STAND UP, and LIE DOWN operated by three volunteers in an indoor environment. In total, the dataset consists of 420 samples of CSI signals with 60 samples for each activity. These time sequence data are composed of 52 subcarriers where the period of each activity determines the length of data ranging from 600 to 1100 sampling measurements. The extracted CSI signals consist of three different types of subcarriers, namely the null subcarriers, the pilot subcarriers, and the data subcarriers. Only the data subcarriers contain crucial information related to human activities among these three types of subcarriers. Therefore, the pilot subcarriers and the Null subcarriers are not utilized for this experimentation following [39].

The second CSI dataset [49], which we called HAR-RT, has been collected based on an Asus RT-AC86U at a frequency band of 80 MHz. HAR-RT consists of six different activities including SIT, STAND, SIT-DOWN, STAND-UP, WALK, and FALL. This indoor CSI information was collected from different spots of the room at different frequency bandwidths in order to avoid possible location and bandwidth dependency. Table 1 shows that the HAR-RT dataset has a total of 1084 samples for six activities with 256 subcarriers where these time sequence data have been normalized.

The third CSI dataset, which we called HAR-ARIL, has been obtained from [50]. This dataset contains six distinct hand activities, namely, hand up, hand down, hand left, hand right, hand circle, and hand cross. The 1440 data samples have been collected based on 15 samples from each activity at 16 different locations by 15 individuals. To ensure data quality, the dataset was manually curated to include 1394 samples.

### 5.2. Experimental Setup

We conducted three experiments to analyse the fusion system for HAR, as shown in Table 2. Under experiment I, various preprocessing parameters were tested with three learning classifiers on the HAR-RP, HAR-RT, and HAR-ARIL datasets in terms of their signal cropping size and the normalized pass-band of the low-pass filter. Under experiment II, various fusion combinations were evaluated on two differently preprocessed data (called process1 and process2) of HAR-RP, HAR-RT, and HAR-ARIL with and without data transformation or normalization. Under experiment III, a comparison between the proposed fusion combination and state-of-the-art (SOTA) methods from [39,49,50] was carried out to observe the accuracy standing of activity recognition.

For the HAR-RP database [39], we randomly selected 336 samples to form the training set and the remaining 84 samples as test samples to follow the 80/20 ratio of a five-fold partitioning. For the HAR-RT database [49], 867 samples were selected to form the training set and the remaining 217 samples were used as test set. For the HAR-ARIL database, 1116 samples were typically used for training and the remaining 278 samples were used for testing. By permuting the above 80/20 ratios, a five-fold cross validation was employed to evaluate the testing accuracy for all the three datasets. All experiments were conducted on a PC equipped with an i9 processor of 3.7 GHz with 32 GB of RAM.

In experiment I, the linear LSE method, the SVM-RBF, and the KNN were utilized as learning classifiers to determine the best combination of the signal cropping size and the normalized pass-band of the low-pass filter. This experiment was conducted in two steps. In step 1, various cropping sizes and normalized pass-bands of the low-pass filter were applied separately to preprocess the CSI signals to determine their desired operating ranges. In step 2, the top two combinations of the cropping size and the normalized pass-band were determined based on the recognition accuracy. These two combinations with top accuracies were named as process1 and process2 respectively.

In experiment II, a score level fusion of the above results was performed using either ANnet, or another LSE classifier, SVM-RBF classifier, or KNN classifier. This experiment was also conducted in two steps. In step 1, process1 and process2 (which were differently preprocessed data as described in above) from experiment I were trained with LSE and SVM-RBF individually in order to generate scores with and without feature transformation/normalization. In step 2, the corresponding scores from different classifier settings were concatenated to form a new set of features for score level fusion using another LSE, SVM-RBF, KNN or ANnet that adopted the training strategy given by (13)–(14).

In experiment III, the proposed score level fusion was compared with SOTA methods in [39,49,50]. The experiment for our fusion was conducted according to the training and test settings in [39,49,50], where the accuracy of activity recognition and processing times were recorded. As for SOTA methods on the HAR-RP dataset, Moshiri et al. [39] converted the CSI data into a 2D array to form a pseudo color image and then utilized a 2D CNN for learning and recognition. Data pre-processing was not applied for the CSI amplitudes since they believed that any extra filtering can result in losing essential information and affect the classification performance. Moreover, the CNN is recommended instead of the LSTM to overcome the high computational complexity and long training time. For the HAR-RT dataset, Schäfer et al. [49] proposed the use of normalized raw CSI data directly in a LSTM network with 100 hidden nodes for HAR. The dataset was randomly divided into 80% for training and 20% for testing. The input dimension for the network was equal to the number of the subcarriers and the dropping rate of the LSTM was set at 40%. As for the HAR-ARIL dataset, the adopted classical machine learning baseline SOTA were the DWT+KNN and SVM-RBF classifiers [50].

### 5.3. Results and Discussion

#### 5.3.1. Experiment I(a) Effect of Cropping Size and Pass-Band on the LSE Classifier

Table 3 shows the impact of cropping size and normalized pass-band on the accuracy of LSE for the HAR-RP, HAR-RT and HAR-ARIL databases. The cropping size refers to the number of effective points of time series data, and the normalized pass-bands refer to the bandwith of the low-pass filter. For the HAR-RP dataset, the result shows that a small crop size leads to a high accuracy at an intermediate range of normalized pass-bands. However, when the normalized pass-band falls below a certain range, the accuracy of LSE drops significantly. For example, at a normalized pass-band of 0.05, the accuracy with cropping size 50 is 40.4%. The result also shows that a combination of cropping size 100 and normalized pass-band 0.05 gives the highest accuracy of 73.6% among all the tested combinations. This is closely followed by the combination of cropping size 100 and pass-band 0.05, which gives an accuracy of 72.8%. For the HAR-RT database, the result shows that accuracy increases as the pass-band value increases. For example, when the cropping size is 50, the accuracy scores for at different pass-band values of 0.1, 0.5, 0.8 and 1.0 are 30.4%, 45.1%, 48.8%, and 71.4%, respectively. The result also shows that the top two accuracy scores are 71.4% and 66.4%, respectively, at cropping sizes 50 and 100. A similar trend in terms of the cropping size and the pass-band is observed for the HAR-ARIL database.

#### 5.3.2. Experiment I(b) to Observe the Effect of Cropping Size and Pass-Band on the SVM-RBF Classifier

As seen from Table 4, the accuracy of SVM-RBF is generally higher than the accuracy of LSE in Experiment I(a). For the HAR-RP database, it can be observed that the accuracy tends to be slightly higher when the cropping size is small. For example, at a cropping size of 50, the accuracy is 96.3% for pass-band 0.1, which is the highest accuracy in the table. However, the second-highest accuracy of 95.6% is achieved at a cropping size of 100 and at pass-band 0.05. This suggests that with a moderate cropping size and pass-band, the SVM-RBF is able to achieve a high level of accuracy. For the HAR-RT database, a similar cropping size pattern is observed at pass-band 1.0, where the top two accuracies of 95.4% and 92.6% are obtained. These values are significantly higher than all other values in the table, which are around 80%. For the HAR-ARIL database, the two best performed results are 72.5% at pass-band 0.3 with cropping size 50 and 73.5% at pass-band 0.5 with cropping size 100.

It is clear that the accuracy is affected by both the cropping size and the pass-band. The patterns of the combination of cropping size and pass-band do show certain common trends between LSE and SVM-RBF. Table 5 shows the chosen parameters, which correspond to the top two accuracies for each of the HAR-RP and HAR-RT datasets.

#### 5.3.3. Experiment I(c) to Observe the Effect of Cropping Size and Pass-Band on the KNN Classifier

According to Table 6, KNN outperforms LSE in terms of accuracy in Experiment I(a), but it is not as accurate as SVM in Experiment I(b). When examining the HAR-RP database, it is clear that accuracy tends to increase slightly with cropping sizes of 50 and 100. For example, the highest accuracy of 92.6% is achieved with a cropping size of 50 and a pass-band of 0.1. The second-highest accuracy of 92.3% is achieved with a cropping size of 100 and a pass-band of 0.05. A similar pattern is observed in the HAR-RT database, where the two highest accuracies of 82.3% and 76.7% are achieved with a pass-band of 1.0 and cropping sizes of 50 and 100, respectively. In the HAR-ARIL database, although performance remains relatively consistent across different cropping sizes and pass-band values, the two highest accuracies are also observed with cropping sizes of 50 and 100. This suggests that KNN can achieve high levels of accuracy with smaller cropping sizes and moderate pass-band values.

#### 5.3.4. Experiment II Fusion of First-Level LSE and SVM-RBF Scores Using LSE, SVM-RBF, KNN, and ANnet

The scores of the first-level LSE and SVM-RBF on the two differently preprocessed data from experiment I are next utilized for fusion under several settings utilizing ANnet, KNN, and another set of LSE and SVM-RBF classifiers. In other words, the LSE and SVM-RBF methods are implemented individually on each preprocessed data (process1 and process2) before fusion with and without transformation/normalization to observe whether the scores from each method are distinguishable. Subsequently, the two individual scores are fused to form a set of new features for final classification decision using LSE, SVM-RBF, KNN, and ANnet. Table 7 shows that by applying a score level fusion method with transformation/normalization, the accuracy of the algorithm is increased in most cases. For the HAR-RP database, the highest accuracy of 97.6% is achieved by applying ANnet and SVM-RBF on the concatenated first-level LSE score of process1 and SVM-RBF score of process2 with transformation. For the HAR-RT database, the highest accuracy of 96.4% is achieved by applying SVM-RBF on the concatenated first-level SVM-RBF score of process1 and SVM-RBF score of process2 with transformation/normalization.

#### 5.3.5. Experiment III Comparison of the Proposed Fusion with SOTA Methods in Table 1

**Figure 4:**
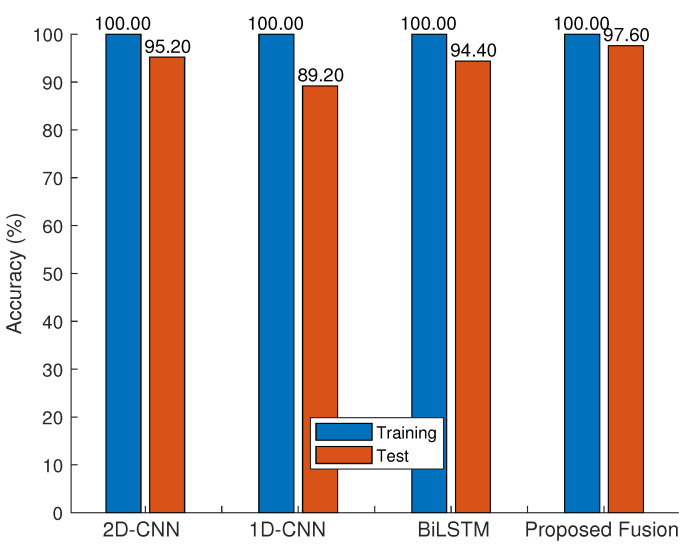
Comparison of our fusion method with methods in Table 1 for the HAR-RP database.

**Figure 5:**
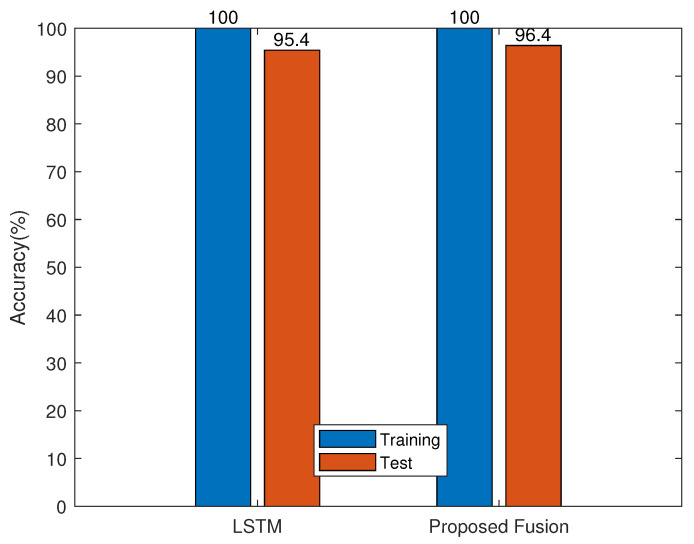
Comparison of our fusion method with methods in Table 1 for the HAR-RT database.

**Figure 6:**
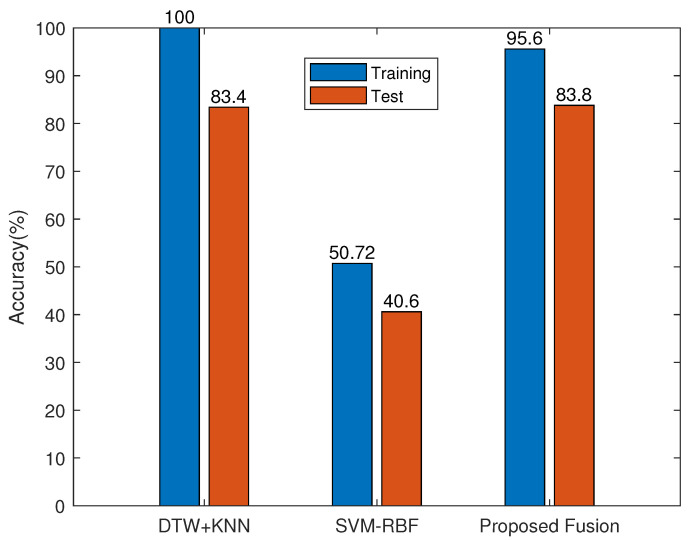
Comparison of our fusion method with methods in Table 1 for the HAR-RT database.

Figure 4 shows the training and test accuracies of the SOTA namely, the 2D CNN, the 1D CNN, the BiLSTM, and the proposed ANnet fusion on the HAR-RP database. The results show significant over-fitting of the SOTA methods compared with that of ANnet. Figure 5 shows the training and test accuracies of the compared algorithms, namely, the LSTM and the proposed ANnet fusion on the HAR-RT database. The results show significant over-fitting of the LSTM compared with ANnet. Figure 6 shows the training and test accuracies of the compared algorithms, namely, the DTW + KNN, SVM-RBF, and the proposed ANnet fusion on the HAR-ARIL database. The results show comparable test accuracies of the proposed ANnet with the classical DTW + KNN method. In terms of the training processing time, our fusion benefits from low computational complexity compared with the deep learning method in Table 8, since our model is a combination of LSE and SVM-RBF with analytic learning.

### 5.4. Summary of Results and Observations

- Expt I: This experiment reveals that the preprocessing steps of selecting the cropping size and the normalized pass-band have a significant impact on the recognition accuracy. In particular, each database shows its best accuracy at different combinations of settings. For example, the HAR-RP and HAR-ARIL datasets show that a small cropping size leads to a high accuracy at an intermediate range of normalized pass-bands. For the HAR-RT database, the accuracy increases as the pass-band value increases.
- Expt II: This experiment shows that fusion using SVM-RBF and ANnet outperforms the LSE and KNN in general. Moreover, many of their fused results show an improved accuracy compared with that before fusion.
- Expt III: This experiment shows that the proposed fusion has either comparable or better accuracy than that of SOTA. In particular, the SOTA methods show significant over-fitting in view of their higher model complexity than the proposed fusion method. In other words, the proposed fusion method has capitalized on the low model complexity but with sufficient mapping capability to generalize the prediction.

## 6. Conclusions

In response to the relatively poor generalization of complex network models on small data sizes, a fusion method with simpler model complexity has been proposed for human activity recognition. This method involves fusing the scores of two first-level classifiers using an analytic network to make the final decision. Experiments have shown not only that this fusion improves recognition accuracy but also that preprocessing of Wi-Fi signals plays a critical role in achieving good baseline recognition accuracy. In particular, varying the cropping size contributes to signal diversity for fusion gain. Additionally, linear LSE and SVM-RBF have been shown to contain complementary information for fusion gain. The proposed simple fusion structure has demonstrated good generalization compared with classical machine learning state-of-the-art methods for the tested datasets.

## Figures and Tables

**Table 1:** Experimental scenarios.

Method	Database	Remark
1D-CNN BiLSTM	HAR-RP: 3 volunteers * 7 activities * 20 samples = 420 samples (sit down, stand up, lie down, run, walk, fall and bend) [39]	The raw CSI amplitude data with 52-dimensional vector.
2D-CNN		CSI signals are converted to RGB images by pseudo color map
LSTM	HAR-RT: 1084 samples with 6 activities (sit, sit down, stand, stand up, walk and fall) [49]	Normalized raw CSI amplitude data with 256-dimensional vector.
DTW+kNN SVM-RBF	HAR-ARIL: 1394 samples with 6 activities (hand up, hand down, hand left, hand right, hand circle, and hand cross) [50]	Normalized raw CSI amplitude data with 52-dimensional vector.

**Table 2:** Experimental setup.

Brief Description of the Experiments	Database
Experiment I Analysis of preprocessing parameters (cropping sizes, filtering band) (a) LSE, (b) SVM, and (c) KNN.	HAR-RP, HAR-RT HAR-ARIL
Experiment II Fusion of first level LSE and SVM-RBF scores using LSE, SVM-RBF, KNN, and ANnet.	HAR-RP, HAR-RT HAR-ARIL
Experiment III Comparison of proposed system with SOTA methods in Table 1.	HAR-RP, HAR-RT HAR-ARIL

**Table 3:** Experiment I(a): recognition accuracies of LSE at various combinations of preprocessing parameters.

Database	Size\Band	0.02	0.05	0.1	0.5
HAR-RP	50	48.1	40.4	**72.8**	72.4
	100	67.3	**73.6**	69.4	68.5
	200	72.5	71.9	69.3	64.5
	500	67.1	67.5	64.2	67.1
Database	Size\Band	0.1	0.5	0.8	1.0
HAR-RT	50	30.4	45.1	48.8	**71.4**
	100	39.1	48.8	50.7	**66.4**
	150	41.9	50.1	52.5	60.3
	200	46.5	51.2	53.5	55.2
Database	Size\Band	0.1	0.3	0.5	0.8
HAR-ARIL	50	37.2	35.4	32.6	42.1
	100	32.1	38.2	48.3	41.6
	150	35.1	48.2	50.1	51.3
	180	43.1	49.2	**51.6**	**51.5**

**Table 4:** Experiment I(b): recognition accuracies of SVM-RBF at various combinations of preprocessing parameters.

Database	Size\Band	0.02	0.05	0.1	0.5
HAR-RP	50	94.2	94.2	**96.3**	95.4
	100	95.1	**95.6**	95.4	95.1
	200	94.5	94.8	94.9	94.5
	500	94.2	94.5	94.6	94.2
Database	Size\Band	0.1	0.5	0.8	1.0
HAR-RT	50	69.5	81.1	81.1	**95.4**
	100	78.8	81.5	81.4	**92.6**
	150	81.1	81.5	81.6	88.0
	200	81.1	81.5	81.6	79.2
Database	Size\Band	0.1	0.3	0.5	0.8
HAR-ARIL	50	66.7	**72.5**	72.1	68.4
	100	68.9	70.3	**73.5**	69.1
	150	69.2	68.2	68.1	69.1
	180	69.5	68.1	68.4	68.7

**Table 5:** Summary of chosen parameters based on Table 3, Table 4, Table 5 and Table 6.

Database	Prepressing	Cropping and Resizing	Normalized Passband
HAR-RP	process1	50	0.1
	process2	100	0.05
HAR-RT	process1	50	1.0
	process2	100	1.0
HAR-ARIL	process1	50	0.3
	process2	100	0.5

**Table 6:** Experiment I(c): recognition accuracies of KNN at various combinations of preprocessing parameters.

Database	Size\Band	0.02	0.05	0.1	0.5
HAR-RP	50	89.2	90.4	**92.6**	90.8
	100	92.1	**92.3**	90.4	91.8
	200	91.6	89.2	92.0	90.6
	500	92.0	90.8	91.8	90.4
Database	Size\Band	0.1	0.5	0.8	1.0
HAR-RT	50	56.7	63.2	72.4	**82.3**
	100	54.5	60.4	68.4	**76.7**
	150	52.3	55.2	52.5	65.1
	200	50.3	54.5	53.5	60.2
Database	Size\Band	0.1	0.3	0.5	0.8
HAR-ARIL	50	63.9	**65.7**	63.8	62.5
	100	62.4	64.5	**64.9**	63.5
	150	63.5	64.1	63.5	63.2
	180	63.1	64.2	63.7	63.9

**Table 7:** Experiment II: performance comparison of fusion methods.

**HAR-RP**						
**Method**	**LSE**	**SVM**	**Score Level Fusion**			
			**LSE**	**SVM**	**KNN**	**ANnet**
W/O transform	process1	process2	52.3	86.9	82.4	88.0
	52.3	95.2				
	process2	process1	69.0	91.7	89.1	90.4
	69.0	94.0				
W transform	process1	process2	92.8	97.6	95.4	97.6
	92.8	96.4				
	process2	process1	94.0	94.0	94.0	95.2
	90.4	94.0				
**HAR-RT**						
**Method**	**SVM**	**SVM**	**Score Level Fusion**			
			**LSE**	**SVM**	**KNN**	**ANnet**
W/O transform	process1	process2	93.5	96.3	94.7	95.4
	95.4	92.6				
W transform	process1	process2	94.5	96.4	95.2	95.4
	95.4	92.6				
**Method**	**LSE**	**SVM**	**Score Level Fusion**			
			**LSE**	**SVM**	**KNN**	**ANnet**
W/O transform	process1	process2	79.2	93.5	90.3	92.6
	71.4	92.6				
	process2	process1	79.7	94.5	91.5	94.0
	66.3	95.4				
W transform	process1	process2	92.1	93.0	92.6	92.6
	76.5	92.6				
	process2	process1	88.9	92.1	89.3	90.8
	76.9	95.4				
**HAR-ARIL**						
**Method**	**SVM**	**SVM**	**Score Level Fusion**			
			**LSE**	**SVM**	**KNN**	**ANnet**
W/O transform	process1	process2	72.5	78.4	74.2	75.5
	72.5	73.5				
W transform	process1	process2	81.7	83.7	82.5	83.8
	80.1	82.3				
**Method**	**KNN**	**SVM**	**Score Level Fusion**			
			**LSE**	**SVM**	**KNN**	**ANnet**
W/O transform	process1	process2	73.6	75.2	74.7	75.2
	65.7	73.5				
	process2	process1	72.5	74.8	73.1	72.6
	64.9	72.5				
W transform	process1	process2	74.3	82.9	82.4	82.5
	70.8	82.3				
	process2	process1	73.0	81.4	80.5	81.5
	69.6	80.1				

**Table 8:** Experiment II: training processing time.

**HAR-RP**	
**Method**	**Execution Time (s)**
ANnet-fusion	15.7
1D-CNN	47.2
2D-CNN	59.8
BiLSTM	67.9
**HAR-RT**	
**Method**	**Execution Time (s)**
ANnet-fusion	58.3
LSTM	376.1
**HAR-ARIL**	
**Method**	**Execution Time (s)**
ANnet-fusion	18.6
DTW+KNN	3356
SVM-RBF	69

## Data Availability

All datasets used in the paper are publicly available.
